# Chromosome-level genome assembly of the sea cucumber *Apostichopus japonicus*

**DOI:** 10.1038/s41597-023-02368-9

**Published:** 2023-07-13

**Authors:** Lina Sun, Chunxi Jiang, Fang Su, Wei Cui, Hongsheng Yang

**Affiliations:** 1 CAS Key Laboratory of Marine Ecology and Environmental Sciences, Institute of Oceanology, Chinese Academy of Sciences, Qingdao, 266071 China; 2 Laboratory for Marine Ecology and Environmental Science, Qingdao National Laboratory for Marine Science and Technology, Qingdao, 266237 China; 3 University of Chinese Academy of Sciences, Beijing, 100049 China

**Keywords:** Genome, Genomics

## Abstract

Sea cucumber is a morphologically diverse and ecologically important clade of echinoderms. The sea cucumber *Apostichopus japonicus* is the most economically valuable species of sea cucumber. The initial assembly of the *A. japonicus* genome was released in 2017. However, this genome assembly is fragmented and lacks relative position information of genes on chromosomes. In this study, we produced a high-quality chromosome-level genome of *A. japonicus* using Pacbio HiFi long-reads and Hi-C sequencing data. The assembled *A. japonicus* genome spanned 671.60 Mb with a contig N50 size of 17.20 Mb and scaffold N50 size of 29.65 Mb. A total of 99.9% of the assembly was anchored to 23 chromosomes. In total, 19,828 genes were annotated, and 97.2% of BUSCO genes were fully represented. This high-quality genome of *A. japonicus* will not only aid in the development of sustainable aquaculture practices, but also lay a foundation for a deeper understanding of their genetic makeup, evolutionary history, and ecological adaptation.

## Background & Summary

The sea cucumber belongs to Echinodermata, which occupy an important phylogenetic position together with their sister phylum, Hemichordata. Evolutionary studies of sea cucumber are crucial for understanding the origin of chordates^1^. Sea cucumbers have evolved special behaviors, including super regenerative capacity, aestivation, and anatomy, among others, to adapt to various oceanic environments. This adaptation has taken place over a long evolutionary history, which can be traced back to the early Cambrian era^2^. Sea cucumbers play crucial roles in maintaining the health of the ocean floor by consuming dead plant and animal matter, which helps to keep the sediment in a balanced state^3^. In addition, sea cucumbers are important economic aquaculture species and have been deemed as one of the most valuable functional foods in the sea due to their nutritional and pharmacological properties^4,5^. Among approximately 60 species with exploitation value, *Apostichopus japonicus* is the most economically important species^6^. In China alone, around 200,000 tons of adult *A. japonicus* are produced each year, with an estimated whole industrial chain value of about 15 billion dollars (Sea cucumber Industry Branch of China Fisheries Association, 2010–2022).

For the reasons listed above, *A. japonicus* is one of the most studied echinoderms, and a total of 1,358 research papers on *A. japonicus* were published between 2000 to 2021^7^. Such research occurs in fisheries^8,9^, immunology^10,11^, food science and biological medicine^4,5^, ecological function^3^, as well as biochemistry and molecular biology^12–14^. With the deepening of research requirements and development of biological technologies, studies of the mechanisms on phenotype formation, physiological responses, and behavioral regulation is rapidly developing. Hence, the genetic resources of *A. japonicus* have been exploited, including the coding and noncoding RNA transcriptome^15–17^, proteome^18^, epigenome^19^, genetic linkage map^20,21^ and genome^1,22^. Among them, the genome is the most basic data that is essential for most multi-omics analyses.

In 2017, Zhang *et al*. constructed the *A. japonicus* genome with the assembly of 805 Mb (contig N50 of 190 Kb and scaffold N50 of 486 Kb)^22^ (Table 1). A total of 30,350 protein-coding genes were annotated in that genome version^22^ (Table 1). In 2018, Li *et al*. published 952 Mb (contig N50 of 45 Kb and scaffold N50 of 196 Kb) of the *A. japonicus* genome with 29,451 protein-coding genes^1^ (Table 1). In 2022, Wang *et al*. constructed a chromosome-level A. japonicus genome using Hi-C technology based on the 2017 genome assembly version^23^. However, the existing genome versions are far behind the growing demand of in-depth study on *A. japonicus* biology. Genomes with a low contig N50 are generally highly fragmented, resulting in the poor annotation of protein-coding genes and non-coding sequences^24^. For example, the sea cucumber breeding industry is currently primarily focused on traditional breeding methods, with molecular marker-assisted breeding playing a supporting role. *A. japonicus* breeding is in the transition from Breeding 2.0 (Statistical and experimental design to improve selection effort) to Breeding 3.0 (Integration of genetic and genomic data)^25^. Genome-wide association studies (GWAS) and genomic selection (GS) are required to identify genes related to economic traits and accurately evaluate them in *A. japonicus* breeding programs. Therefore, genomes with long contig N50s and long continuity are essential. Moreover, the annotation of functional genes and regulatory elements plays important roles in understanding evolutionary mechanisms and genetic regulation, which depend on the high quality of genome.

**Table 1:** Comparison of *A. japonicus* genome assemblies in 2017, 2018 and 2023.

Year	2017	2018	2023
Sequencing instrument	Illumina + PacBio (CLR)	Illumina + PacBio (CLR)	Illumina + Pacbio (CCS)
# Reads	128,784,478	—	10,588,443
# Bases (Gbp)	260 (Illumina) + 64 (Pacbio)	349 (Illumina) + 23 (Pacbio)	56.54 (Illumina) + 43.22 (Pacbio)
Coverage	295 × (Illumina) + 73 × (Pacbio)	346 × (Illumina) + 24 × (Pacbio)	84 × (Illumina) + 64 × (Pacbio)
Hi-C(Gbp)	—	—	3.18
# Chromosomes	—	22	23
Genome size (Mbp)	804.9	952	671.6
# Contigs	4,741	21,303	198
Contig N50 (Mbp)	0.190	0.045	17.20
# Scaffolds	3,281	7,286	34
Scaffold N50 (Mbp)	0.486	0.196	29.65
Repeat rate (%)	27.2	26.6	47.33
GC content (%)	36.75	37.37	38.56
# Genes	30,350	29,451	19,828
miRNAs	137	—	1,066
tRNAs	1,127	—	4,963
rRNAs	75	—	3,379
snRNAs	223	—	1,088

**Fig. 1:**
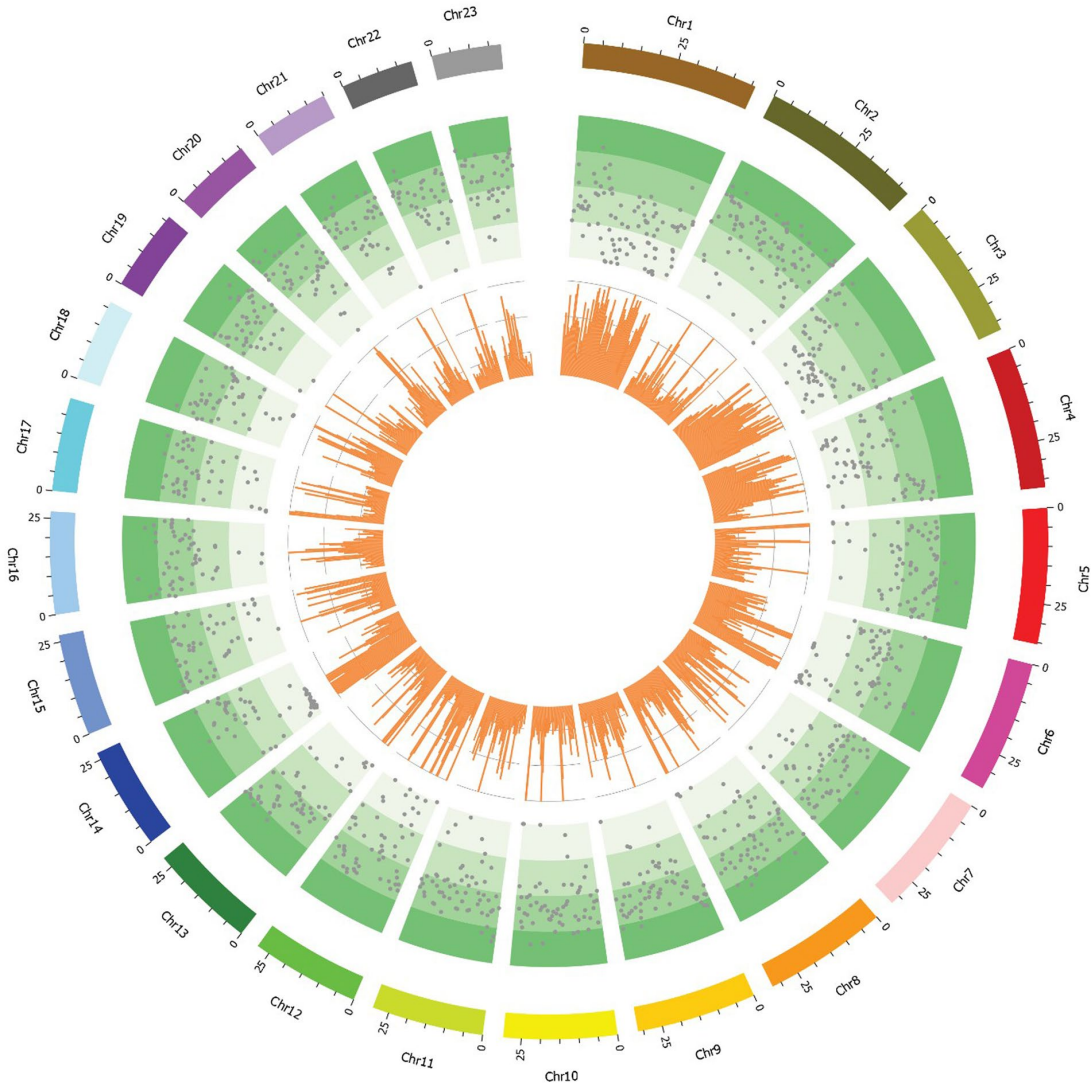
A circos plot of 23 chromosomes of *A. japonicus* genome. The tracks from inside to outside are: bar plot for gene density profile, the distributions of transposable element and 23 chromosomes.

We applied multiple sequencing technologies, generating 56.54 Gb of Illumina data, 43.22 Gb of PacBio data, and 3.18 Gb of HiC data, to reconstruct the chromosome-level *A. japonicus* genome (Fig. 1), which is the first known chromosome-level genome of sea cucumber (Table 1). The final assembly was 671.60 Mb in total length with a contig N50 length of 17.20 Mb and scaffold N50 length of 29.65 Mb (Table 1). The assembly quality was much better than those of the previous genome versions. The genome developed herein will be an excellent tool to better investigate the mechanisms that drive evolution and biodiversity^23^. By analyzing their genetic basis, researchers can identify the compounds responsible for these benefits and develop new treatments for human diseases^4^. Moreover, scientists can better understand the genetic basis of their responses to environmental stressors by studying their genomes, and develop new tools for monitoring and conserving the ocean’s resources^26^. As an important aquaculture species, the accurate genetic analysis of economic traits can help to improve the genetic stability of target traits and the success rate of genetic improvement operations^20^. Overall, constructing a high-quality genome for sea cucumbers is a crucial step in advancing the understanding of these unique animals and their importance to the health of the ocean and to human well-being.

## Methods

### Sample collection and sequencing

The longitudinal muscle of a female *A. japonicus* was collected in Rushan, Shandong Province, China, in 2021. The sample was washed three times with phosphate buffered saline (PBS), quickly frozen in liquid nitrogen, and stored at −80 °C until DNA extraction. After DNA extraction, a short fragmented library was prepared with an insert size of 350 bp and sequenced using the Illumina Platform to generate 150-bp paired-end reads. For HiFi read generation, high-molecular-weight (HMW) gDNA was sheared to approximately 15 Kb before preparing a PacBio HiFi library. The genomic library was sequenced in CCS mode on the PacBio Sequel II system at Novogene (Beijing, China). After trimming the low-quality reads and adaptor sequences from the raw data, 56.54 Gb of Illumina data and 43.22 Gb of PacBio data with a mean read length of 14.7 Kb were obtained, resulting in 83.58-fold and 63.89-fold coverage of the *A. japonicus* genome respectively (Table 2). Such coverage was sufficient for haplotype-resolved assembly.

**Table 2:** Statistical analysis of sequencing reads from Illumina and PacBio.

libraries	Total data (G)	Read length (bp)	Sequence coverage (X)
Illumina reads	56.54	150	83.58
PacBio reads	43.22	14,729 (mean)	63.89
Total	99.76	—	147.47

### Genome assembly

The genome was assembled using the default parameters of Hifiasm (v0.15.4-r343)^27^. Hifiasm calculates from the uncollapsed genome, allowing it to preserve haplotype information as much as possible. The HiFi long reads were provided to Hifiasm to generate the monoploid and a pair of haplotype-resolved assembly contig graphs. We assembled 178 contigs with a total length of 671.63 Mb. The maximum contig size and N50 were 38.08 and 17.20 Mb (Table 3), respectively.

**Table 3:** Assembly statistics at the contig level.

Type	Contig (bp)
Total Number	178
Total Length	671,627,515
Average Length	3,773,188
Max Length	38,087,046
Min Length	18,165
N50 Length	17,200,168
N50 Number	14
N90 Length	9,296,432
N90 Number	35

### Hi-C library preparation, sequencing, and chromosome anchoring

A Hi-C library was prepared following the Hi-C library protocol^28^. After grinding with liquid nitrogen, fresh muscle was cross-linked using 4% formaldehyde solution at room temperature in a vacuum for 30 min. The fixation was terminated using 2.5 M glycine. Following cell lysis, cross-linked DNA was digested using the four-cutter restriction enzyme MboI. The DNA ends were subsequently labeled with biotin-14-dCTP and subjected to blunt-end ligation of the cross-linked fragments. DNA was extracted and purified using the phenol-chloroform extraction method. Sonication was employed to generate fragments ranging from 200 to 600 base pairs, and the ends of these fragments were repaired using a combination of T4 DNA polymerase, T4 polynucleotide kinase, and Klenow DNA polymerase. Streptavidin C1 magnetic beads were utilized for the specific enrichment of biotin-labeled Hi-C samples^29,30^. Following the addition of A-tails to the fragment ends and ligation with Illumina paired-end (PE) sequencing adapters, Hi-C sequencing libraries were subjected to PCR amplification (12–14 cycles) and subsequently sequenced on an Illumina PE150 platform. The raw sequence data were filtered to obtain a total of 73.50 Gb of clean data, with Q20 = 96.28% and Q30 = 91.25% (Table 4), which was used to assist chromosome assembly.

**Table 4:** Statistical analysis of sequencing data from Hi-C.

Type	Data
Raw paired reads	244,997,475
Raw Base(bp)	73,499,242,500
Clean Base(bp)	73,002,860,400
Effective Rate(%)	99.28
Q20(%)	96.28
Q30(%)	91.25
GC Content(%)	38.56

**Fig. 2:**
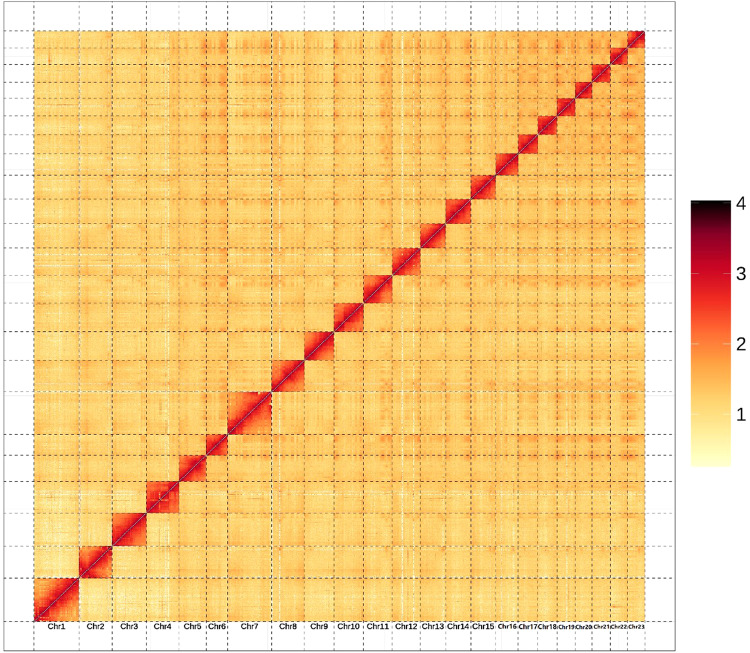
Genome-wide Hi-C heatmap of *Apostichopus japonicus*.

HiCUP (v0.8.1) was used to process the Hi-C data^31^. The clean Hi-C data was assembled using the ALLHiC pipeline, which contained a total of five steps: pruning, partitioning, rescuing, optimizing and building^32,33^. Finally, 99.94% of the initial assembled sequences were anchored to 23 pseudo-chromosomes (Fig. 2) with lengths ranging from 18.21 to 46.02 Mb. The total length of the genome assembly was 671.63 Mb, with 34 scaffolds and a scaffold N50 of 29.65 Mb (Table 5).

**Table 5:** Assembly statistics for Hi-C.

Type	Contig length	Scaffold length	Contig number	Scaffold number
Total	671,627,515	671,643,915	198	34
N50	15,848,779	29,647,521	15	10
N90	8,359,816	20,397,903	38	20
Place	—	671,222,061	—	23
Unplace	—	421,854	—	11

### Genomic repeat annotation and ncRNA annotation

**Fig. 3:**
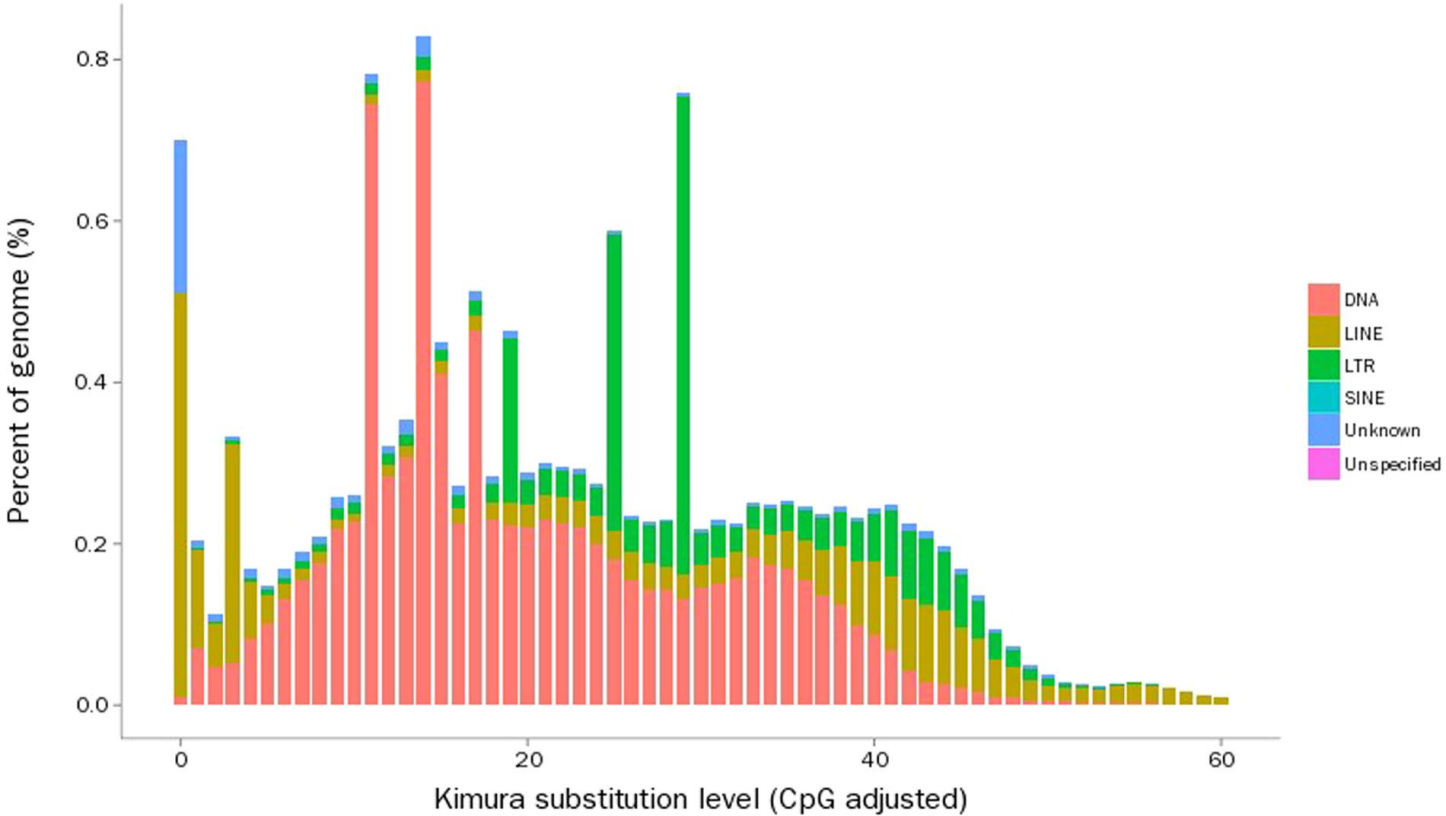
Distribution of divergence rates for TEs in the *A. japonicus* genome.

Repeat sequences of the *A. japonicus* genome were identified by homology-based and *de novo* strategies^34^. First, we integrated the repetitive sequence database predicted by Denovo with the homologous repetitive sequence database, Repbase^35^. Then, we used RepeatScout (v1.0.5)^34^, RepeatModeler (v2.0.1)^36^, Piler (v1.0)^37^ and LTR-FINDER (v1.0.6)^38^ to identify transposable element (TE) families. Repeatmasker (v4.1.0)^36^, RepeatProteinMask (v4.1.0) and TRF (v4.0.9)^39^ were used to identify and classify different repetitive elements by aligning the *A. japonicus* genome sequences against the integrated database. After removing the redundancy results obtained using the above three methods, the total length of the repeat sequences accounted for 47.33% of the *A. japonicus* genome. In addition, the Kimura divergence value of TE was calculated using calcDivergenceFromalign.pl^40^. TE landscapes were drawn using createRepeatLandscape.pl^41^ (Fig. 3). Among the repeat elements, short interspersed nuclear elements (SINEs) accounted for 0.02% of the genome and long interspersed nuclear elements (LINEs) accounted for 2.94% of the genome. Long terminal repeats (LTRs) and DNA elements accounted for 27.03% and 3.74% of the genome, respectively (Table 6).

**Table 6:** Classification of repetitive sequences and ncRNAs in the *A. japonicus* genome.

Type		Denovo + Repbase		TE Proteins		Combined TEs	
		Length(bp)	% in Genome	Length(bp)	% in Genome	Length(bp)	% in Genome
DNA		24,861,559	3.70	517,042	0.08	25,141,601	3.74
LINE		16,671,289	2.48	5,258,345	0.78	19,744,382	2.94
SINE		159,644	0.02	0	0	159,644	0.02
LTR		181,153,677	26.97	4,991,945	0.74	181,560,277	27.03
Unknown		78,711,126	11.72	0	0	78,711,126	11.72
Total		287,263,851	42.77	10,766,642	1.60	288,599,322	42.97
**Type**		**Copy number**		**Total length(bp)**		**% of genome**	
miRNA		1,066		117,207		0.017451	
tRNA		4,963		367,482		0.054714	
rRNA	rRNA	3,379		421,316		0.062729	
	18 S	558		130,190		0.019384	
	28 S	703		168,303		0.025058	
	5.8 S	86		7,311		0.001089	
	5 S	2,032		115,512		0.017198	
snRNA	snRNA	1,088		142,445		0.021208	
	CD-box	70		10,703		0.001594	
	HACA-box	12		2,126		0.000317	
	splicing	996		127,438		0.018974	
	scaRNA	4		661		0.000098	
	Unknown	6		1,517		0.00022	

For the annotation of noncoding RNA (ncRNA), tRNAScan (v1.4)^42^ and blast (v2.2.26)^43^ were used for tRNA and rRNA prediction, respectively. Other noncoding RNAs, including miRNA and snRNA were detected by alignment to the Rfam database^44^ using INFERNAL (v1.0)^45^. Four types of noncoding RNAs, including 1,066 miRNAs, 4,963 tRNAs, 3,379 rRNAs, and 1,088 snRNAs, were identified from the *A. japonicus* genome (Table 6).

### Protein-coding gene prediction and annotation

**Fig. 4:**
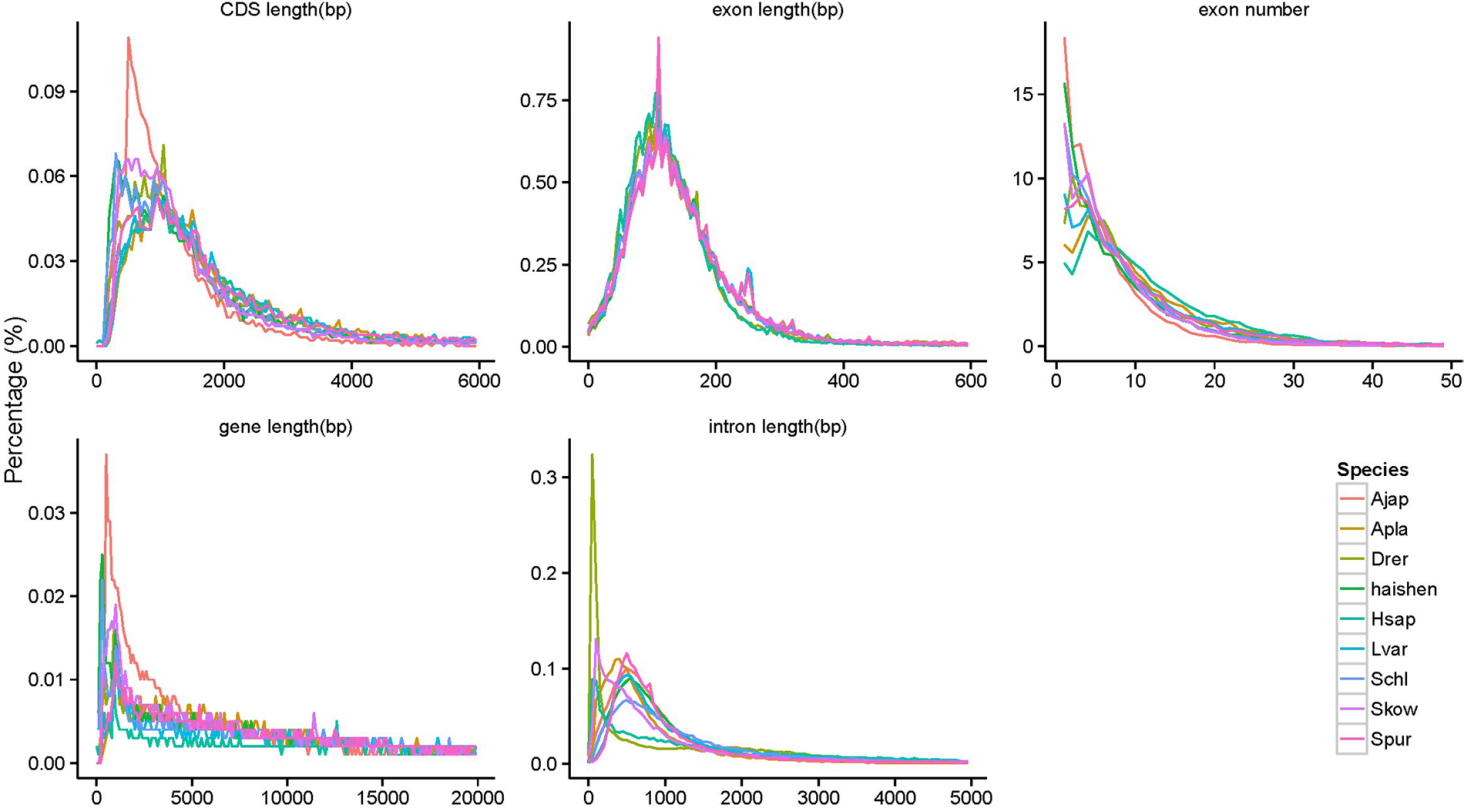
Comparisons of the genomic elements of closely related species.

Gene structures were predicted using three basic strategies: *de novo*, homology-based, and transcriptome sequencing-based prediction. Based on the genome sequence, we used Augustus (v3.2.3)^46^, GlimmerHMM (v3.0.4)^47^, SNAP (v2013.11.29)^48^, Geneid (v1.4)^49^ and Genscan (v1.0)^50^ for ab initio gene prediction. For homology-based gene prediction, the protein sequences of *L. variegatus*^51^, *S. purpuratus*^52^, *A. planci*^53^, *H. sapiens*^54^, *D. rerio*^55^, *S. chloronotus*^56^ and *S. kowalevskii*^57^ were downloaded from the National Center for Biotechnology Information (NCBI). Blast (v2.2.26)^43^ and Genewise (v2.4.1)^58^ were used to align the protein sequences of *A. japonicus* to the seven other species for homology-based gene prediction. A total of 10,707, 11,249, 9,982, 7,184, 7,374, 15,566 and 11,377 genes were identified for *L. variegatus*, *S. purpuratus*, *A. planci*, *H. sapiens*, *D. rerio*, *S. chloronotus* and *S. kowalevskii*, respectively (Table 7). We also compared the gene, CDS, and exon and intron lengths to those of the seven other species (Fig. 4). For *A. japonicus*, the average lengths of the transcript, exons, and introns were 7,736.67, 193.65, and 1,286.06 bp, respectively.

**Table 7:** Statistical analyses of the gene structure annotation of the *A. japonicus* genome.

	Gene set	Number	Average transcript length(bp)	Average CDS length(bp)	Average exon length(bp)	Average intron length(bp)
*De novo*	Augustus	19,684	12,358.41	1,546.20	7.79	1,593.00
	GlimmerHMM	44,287	13,553.26	795.90	4.88	3,290.80
	SNAP	26,032	25,835.92	1,012.99	6.55	4,473.70
	Geneid	17,957	21,083.14	1,702.56	7.69	2,896.45
	Genscan	18,774	23,593.41	1,770.98	8.75	2,816.45
Homolog	Skow	11,377	6,900.30	6,900.30	4.78	1,566.05
	Ajap	19,702	7,316.82	7,316.82	5.86	1,277.02
	Hsap	7,184	8,555.73	8,555.73	5.82	1,549.13
	Apla	9,982	10,725.65	10,725.65	6.86	1,600.22
	Spur	11,249	9,002.69	9,002.69	6.22	1,486.67
	Schl	15,566	11,840.35	11,840.35	7.31	1,645.92
	Lvar	10,707	9,336.09	9,336.09	6.48	1,467.80
	Drer	7,374	8,030.81	8,030.81	5.49	1,547.84
RNAseq	PASA	18,773	17,353.65	17,353.65	7.13	2,615.21
	Transcripts	27,221	33,433.24	33,433.24	8.85	3,775.72
EVM		19,961	14,204.95	14,204.95	7.96	1,817.50
Pasa-update*		19,850	14,577.36	14,577.36	8.05	1,844.83
Final set*		19,827	14,590.03	14,590.03	8.06	1,844.91
Final set update*		19,828	14,589.57	14,589.57	8.06	1,844.92

**Fig. 5:**
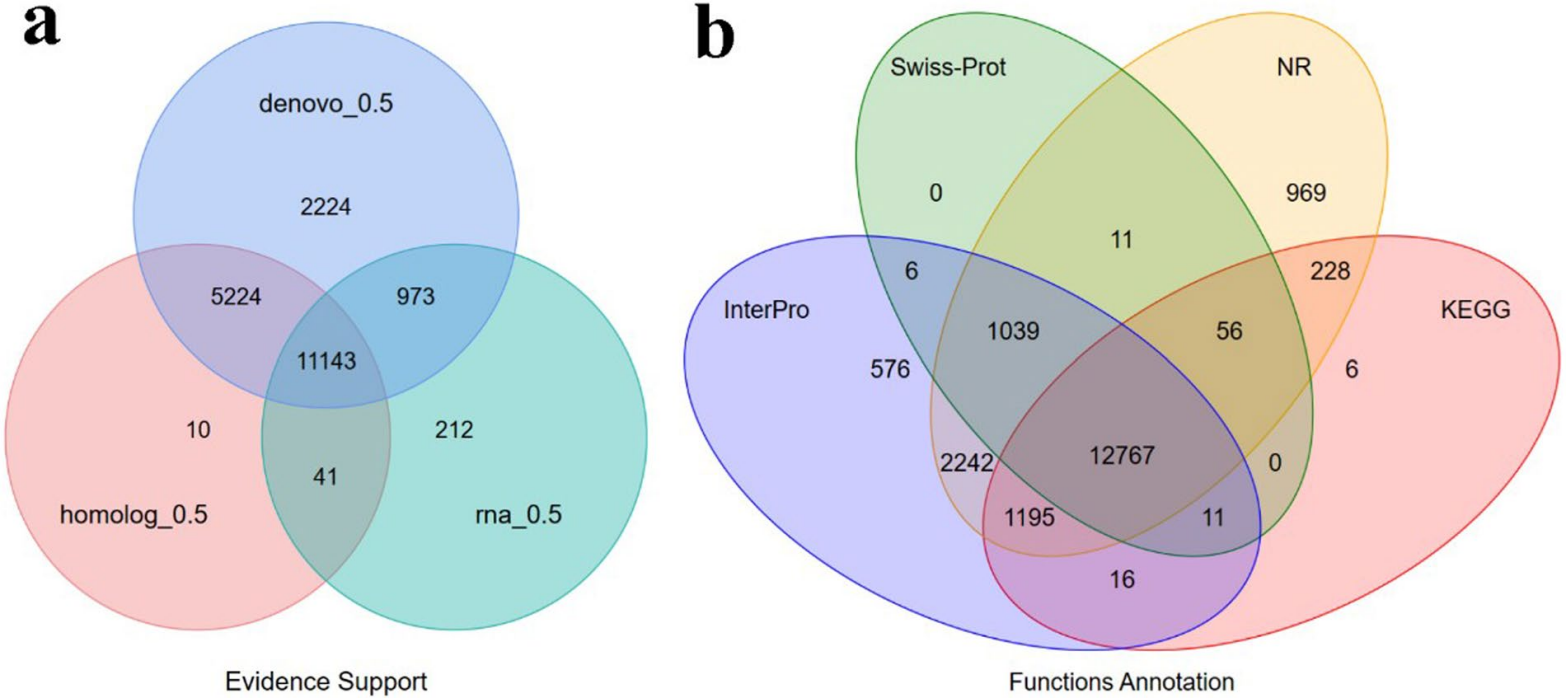
Gene prediction and functional annotation of the *A. japonicus* genome. (**a**) Venn diagram of the gene set prediction. (**b**) Venn diagram of functional annotation based on different databases.

The clean RNA-seq data underwent two types of assembly methods. For transcript assembly, we relied on the reference genome, while *de novo* assembly was carried out using Trinity (v2.11.0)^59^. Open reading frames (ORFs) were detected using PASA (v2.1.0)^60^. Based on the predictions, we used EvidenceModeler(v1.1.1)^61^ to integrate the gene sets predicted using different strategies into a non-redundant and complete gene set of 19,828 protein-coding genes (Table 7 & Fig. 5a).

To perform functional annotation of protein-coding genes, Blastp (v2.2.26)^62^ and Diamond (v0.8.22)^63^ were used to align protein-coding genes to the SwissProt^64^, NCBI Non-redundant protein(NR) (ftp://ftp.ncbi.nih.gov/pub/nrdb/), KEGG^65^, InterPro^66^, GO Ontology (GO)^67^ and Pfam^68^ protein databases using an E-value threshold of 1E-5. The protein domains and motifs were annotated using InterProScan (v5.52-86.0)^69^. Finally, 19,122 (96.40%) of the 19828 predicted genes were annotated by at least one database (Table 8). Of the functional proteins, 12,767 (64.39%) were supported by all four databases (Fig. 5b).

**Table 8:** Statistical analysis of the functional gene annotations of the *A. japonicus* genome.

Database	Number	Percent(%)
Total	19,828	—
Swissprot	13,890	70.10
Nr	18,507	93.30
KEGG	14,279	72.00
InterPro	17,852	90.00
GO	10,781	54.40
Pfam	13,332	67.20
Annotated	19,122	96.40
Unannotated	706	3.60

## Data Records

The genomic Illumina sequencing data were deposited in the SRA at NCBI SRR22523578^70^.

The genomic PacBio sequencing data were deposited in the SRA at NCBI SRR22799261^71^, SRR23640106^72^-SRR23640107^73^.

The transcriptomic sequencing data were deposited in the SRA at NCBI SRR17056084^74^.

The Hi-C sequencing data were deposited in the SRA at NCBI SRR23362389- SRR23362392^75–78^.

The final chromosome assembly and genome annotation files are available in Figshare^79^.

## Technical Validation

### Evaluation of the genome assembly and annotation

We evaluated the genome assembly quality through the following measures: (i) The BUSCO (V4.1.2)^80^ evaluation was performed using a single-copy orthologous gene library, combined with software tools such as tblastn, augustus, and hmmer, to assess the assembled genome. The result showed that 97.2% of gene orthologs were detected in *A. japonicus*. Among them, 96.7% achieved complete scores, while 0.5% obtained fragment scores. This indicates a relatively comprehensive assembly outcome (Supplementary fig. 1). (ii) Employing the Core Eukaryotic Genes Mapping Approach (CEGMA) (v2.5)^81^, we identified 458 core eukaryotic genes, including 248 highly-conserved core genes used to assess genome and annotation completeness (Supplementary table 1). By aligning *A. japonicus* genes to these 248 core genes, we observed homologous genes in the *A. japonicus* gene sets for 228 core genes, accounting for 91.94% of the total. These findings further support the relatively complete assembly results. (iii) By aligning Illumina sequencing reads to the nuclear genome using BWA (v0.7.8)^82^, we determined a read mapping rate of 96.86% and a coverage rate of 99.83%, indicating high mapping efficiency and comprehensive coverage. (iv) The consensus quality value (QV) of genomes representing per-base consensus accuracy was estimated by Merqury^83^, and the QV of the *A. japonicus* genome exceeded 45 (48.86), which indicated the high accuracy of the genome assembly. Thus, all of the above results indicated that we obtained the high-quality genome of *A. japonicus*.

## Supplementary information

Supplementary Material

## Data Availability

No custom scripts or code were used.
