# Supplementary material for: Chromosome-level genome assembly of the sea cucumber Apostichopus japonicus
Source: Sci Data. 2023 Jul 13;10:454. doi: 10.1038/s41597-023-02368-9 (PMC10344927; doi:10.1038/s41597-023-02368-9)
Supplement: Supplementary file 1 — Supplementary Material [file 41597_2023_2368_MOESM1_ESM.docx]

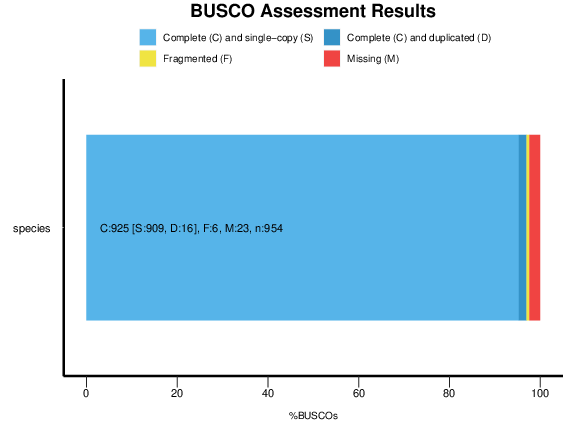


Supplementary figure 1. BUSCO assessment results

Supplementary table 1. Genome CEGMA evaluation results.

| **Species** | **Complete** | | **Complete + Partial** | |
| --- | --- | --- | --- | --- |
|  | **# Prots** | **%Completeness** | **# Prots** | **%Completeness** |
| *Apostichopus japonicus* | 227 | 91.53 | 230 | 92.74 |
